# Implementing emerging customer screening standards for nucleic acid synthesis

**DOI:** 10.3389/fbioe.2026.1819810

**Published:** 2026-07-29

**Authors:** Sarah R. Carter, Lucas Boldrini, Tessa Alexanian

**Affiliations:** 1 Science Policy Consulting LLC, Arlington, VA, United States; 2 International Biosecurity and Biosafety Initiative for Science (IBBIS), Geneva, Switzerland

**Keywords:** biosecurity, biosecurity policy, customer screening, know your customer (KYC), nucleic acid synthesis, standards development, synthesis screening

## Abstract

The decision to fulfill an order for synthetic nucleic acids hinges on whether the customer can be trusted to receive them. A consensus on customer screening practices that allow synthesis providers to make that decision has begun to emerge. In 2024–2025, international and national-level guidance aligned on five practices: tiered screening, with more rigorous screening for orders containing sequences of concern (SOCs); identity verification for all customers; legitimacy verification for customers ordering SOCs; legitimate use verification for SOCs; and supply chain screening to address third-party vendors and multi-party distribution. Translating this emerging consensus into practical tools, however, reveals persistent ambiguities about appropriate due diligence and decision-making. Drawing on experience developing standardized customer screening forms, this paper describes the emerging consensus on best practices, analyzes implementation challenges, and recommends five projects to strengthen customer screening: establishing a globally-relevant baseline of customer attributes that should trigger additional scrutiny; clarifying ambiguities through geographically diverse case studies; encouraging automated identity verification; leveraging existing institutional and government approvals to demonstrate legitimacy; and integrating sequence and customer screening across standardized risk tiers.

## 1 Introduction

A consensus is emerging on appropriate customer screening practices for providers of synthetic nucleic acids. In 2024 and 2025, several analyses explored practices and challenges particular to customer screening (Alexanian and Carter, 2024; Carter, 2024; Crawford et al., 2024; Alexanian and Nevo, 2024; Engineering Biology Research Consortium, 2025b), and international bodies and national governments issued guidance to support customer screening practices. These new policies include an international standard for nucleic acid synthesis (International Organization for Standardization, 2024), guidance from the U.K. Department of Science, Innovation and Technology (UK Department for Science, Innovation, and Technology, 2024), and an updated voluntary protocol from the International Gene Synthesis Consortium (International Gene Synthesis Consortium, 2024). New requirements were also established by the U.S. Office of Science and Technology Policy (Office of Science and Technology Policy, 2024), and, although the policy is under review by the current administration (The White House, 2025), we anticipate that its core features will be maintained.

A comparison of these new policies shows alignment across five key areas.

1. Tiered screening should implement baseline screening for all orders of synthetic nucleic acids, and incorporate additional follow-up screening when orders include sequences of concern (SOCs) that could facilitate the construction of dangerous biological agents.
2. Identity verification should be performed for all customers of synthetic nucleic acids.
3. Legitimacy verification should be performed for customers who order SOCs.
4. Legitimate use of the ordered products should be verified for orders that include SOCs.
5. Supply chain screening should consider that providers do not necessarily sell directly to the individual who will use the ordered sequences. Guidance now defines customer and end user separately and specifies screening responsibilities for non-traditional providers and third-party vendors.

Table 1 shows specific practices recommended by these policies, demonstrating substantial alignment. A compilation of guidance on customer screening from each of the four policies referenced is available in the Supplementary Material.

**TABLE 1:** Emerging consensus in customer screening across four policies issued in 2024, including the U.S. OSTP Framework for Nucleic Acid Synthesis Screening (OSTP), the U.K. DSIT Screening guidance on synthetic nucleic acids for users and providers (UK), the IGSC Harmonized Screening Protocol v3.0 (IGSC), and ISO 20688–2:2024 (ISO).

Customer screening practices	OSTP	UK	IGSC	ISO
Tiered Screening				
Screen customers for all orders	✓	✓	✓	✓
Screen customers based on sequence analysis	✓	✓	✓	✓
Conduct baseline screening during onboarding	✓	✓	✓	✓
Conduct additional screening for sequences of concern (SOCs)	✓	✓	✓	•
Customer Identity Verification				
Verify customer identity for all orders	✓	✓	✓	✓
Collect basic information (name, organization, contact)	✓	✓	✓	✓
Perform due diligence for all orders (e.g., sanctions lists)	✓	✓	✓	•
For SOCs, conduct enhanced identity verification	✓	✓	✓	•
For SOCs, verify identity using government-issued ID	X	✓	✓	X
Use automated tools and digital identity standards	✓	X	•	X
Check shipping address for consistency and legitimacy	•	•	•	•
Customer Legitimacy Verification				
Verify customer legitimacy for SOCs	✓	✓	✓	✓
Check institutional affiliation and institutional legitimacy	✓	✓	✓	✓
Check individual research history (e.g., ORCID, publications)	✓	✓	•	✓
Check institution’s mission or purpose relates to life sciences	✓	•	✓	•
Verify government-issued permits to possess SOCs	✓	✓	•	X
Request institutional biosafety officer/oversight information	✓	✓	X	X
Verify institutional legal status (e.g., incorporation, licenses)	✓	•	•	•
Legitimate Use Verification				
Verify legitimate use for SOCs	✓	✓	✓	✓
Request written description of intended product use for SOCs	✓	✓	✓	✓
Include export control declaration and resolve licensing needs	✓	✓	✓	X
Use institutional approval process (e.g., biosafety officer sign-off)	✓	•	•	•
Evaluate in the context of research history or business plans	•	•	•	X
Apply more stringent checks if no institutional oversight exists	•	•	X	X
Supply Chain Screening				
Identify whether customer is the end user or a third party	✓	✓	✓	•
For third parties, verify institutional legitimacy with documentation	✓	✓	✓	•
Require third-party vendors attest to verifying end user identity, affiliation, and use	✓	✓	✓	•

Each practice is coded by whether the policy explicitly mentions or requires it ( ✓ ), partially includes or implies it (•) , or does not explicitly mention it (X).

Despite the growing consensus on customer screening, there are persistent ambiguities around appropriate due diligence and decision-making when implementing these practices. For example, what constitutes an acceptable level of identity verification for a new customer ordering benign DNA? Is a valid email address adequate? Is it ever appropriate to ship orders to a residential address? What type of documentation is acceptable to demonstrate that a newly-established company has a legitimate use for SOCs? To what extent should a provider trust information provided by the customer?

Some differences in decision making are unavoidable and reflect the international and institutional diversity of nucleic acid providers and customers, risk tolerances, familiarity of nucleic acid providers with different types of customers, and the broader context for each order. However, in many cases, additional resources and tools could support best practices. Below, we discuss areas of consensus and remaining challenges for implementing customer screening in the context of nucleic acid synthesis. For each area, we provide perspective by highlighting the experience of the International Biosecurity and Biosafety Initiative for Science (IBBIS) in developing its openly available customer screening forms and guidance. IBBIS is a non-profit organization based in Geneva that supports nucleic acid synthesis screening globally. In many cases, additional resources and tools could support best practices for customer screening across the community, and we provide recommendations to advance this work.

## 2 Implementing emerging standards in customer screening

Drawing on the points of consensus listed above, extensive outreach with the community of nucleic acid providers (Alexanian and Carter, 2024), and input and feedback on best practices (Engineering Biology Research Consortium, 2025b), IBBIS has developed openly available forms and decision guidance to support tiered screening of customers by nucleic acid synthesis providers (International Biosecurity and Biosafety Initiative for Science IBBIS, 2024). The New Customer Form collects basic information to verify customer identity for each new customer and to determine whether they are part of a multi-party supply chain. The SOC Order Form collects information needed to verify customer legitimacy and legitimate use. To support decision making by synthesis providers, IBBIS has also developed Decision Support guidance with step-by-step instructions for how the information in these forms should be evaluated. Here, we describe how IBBIS has grappled with each of the five areas of emerging consensus on customer screening, and highlight remaining challenges and broader considerations.

### 2.1 Tiered screening

Current guidance recommends a baseline level of screening for all customers, generally conducted during customer onboarding (i.e., the first time a customer makes an order with a provider), as well as a higher level of screening or “follow-up screening” when a customer orders a sequence of concern.

With its “New Customer Form” and “SOC Order Form,” IBBIS implemented a tiered approach to ensure that the majority of customers, who neither order SOCs nor act as third-party vendors, face minimal friction. To account for different levels of risk that different SOCs may pose, decision support guidance for the SOC Order Form includes options at different levels of due diligence for verifying customer identity, customer legitimacy, and institutional oversight.

These two tiers (“all orders” and “orders containing SOCs”) may not be sufficient to reflect the risk from synthesis orders. The IGSC Harmonized Protocol highlights orders that include “significant portions of the genomes from regulated pathogens or toxins” for additional scrutiny (International Gene Synthesis Consortium, 2024, p. 15), and all guidance related to nucleic acid synthesis screening recommends that providers pay particular attention to regulated sequences. However, regulatory controls vary jurisdictions and in some cases there is ambiguity about which SOCs are regulated. Programs such as the UK Specified Animal Pathogens Order (SAPO) and US Federal Select Agents Program (FSAP) require government-issued licenses before researchers can acquire certain pathogens, but their application to nucleic acids, particularly sequences that only partially encode proteins, is not always clear. Export control guidance, such as that issued by the Australia Group (followed by 42 countries and the European Union), has similar ambiguity: controls on nucleic acid sequences that “endow or enhance pathogenicity” (The Australia Group, 2023) do not clearly delineate which sequences meet this standard. Connecting pathogen-level control lists to sequence risk evaluation is under active investigation (Alexanian et al., 2026), but outside the scope of this paper. At present, some sequence screening tools offer multiple tiers of SOCs (Table 2), but these risk tiers are not standardized across tools or jurisdictions, and still require specialized expertise in dual-use research to translate into customer screening decisions.

**TABLE 2:** Multiple sequence screening tools incorporate risk tiers beyond “flag” or “no flag” as a sequence of concern.

Tool	Sequence risk tiers
Aclid	SOCs may be flagged as higher concern (based on regulation by different biosecurity frameworks) or lower concern (if known to have a metabolic or housekeeping function) (Flyangolts, 2025)
commec (IBBIS)	SOCs may be flagged as higher concern (due to matching a curated biorisk database) or lower concern (due to matching a curated benign database) (Wheeler et al., 2024)
SecureDNA	SOCs may be flagged as higher concern (due to matching an organism with known pandemic potential, or known Arthropod-to-Human or Human-to-Human transmission) or lower concern (e.g., matching against non-pathogenic or non-toxin-producing genes) (Baum et al., 2026)
UltraSEQ (Battelle)	SOCs are automatically sorted into five risk tiers, ranging from highest concern (toxins, viruses or SOCs that transfer virulence from Tier 1 Agents) to lowest concern (SOCs from non-Select Agents) (Gemler et al., 2024)

### 2.2 Customer identity verification

There is broad agreement that nucleic acid providers should verify their customer’s identity, but questions remain about the level of due diligence that a provider should conduct to ensure that the customer is being truthful.

For all customers, the IBBIS New Customer Form requests basic identifying information: name, email, phone number, shipping address, institutional affiliation, and website. This is consistent with the information many providers already request, which is collected in part because it is useful when fulfilling orders. The decision guidance calls for verifying this information with “some” due diligence, including checking the individual’s name against government watchlists, verifying contact information (i.e., that the email address can be used to successfully contact the customer), checking that the email address extension matches the institution or organization listed, and verifying shipping addresses using mapping tools.

Although these checks are considered best practices, government watchlists can be difficult to use successfully and email-based checks are unreliable. A report on global sanctions found that most countries do not have an autonomous sanctions list and few have a well-structured, easy-to-find list that providers could reference (Castellum AI, 2024). Even if a list is found, verifying sanctions flags can be challenging because of name collisions and other ambiguities, especially when handling non-Latin scripts. Email-based checks also face ambiguities; personal communication with providers indicates that legitimate researchers in parts of Sub-Saharan Africa often use personal email addresses even when they have an affiliation with a well-established university.

These limitations are even more pronounced when nucleic acid providers are expected to verify customer identity with a higher level of due diligence for customers who order sequences of concern. IBBIS guidance for SOC orders calls for verification of this basic information with “enhanced” due diligence, which might be achieved through real-time contact (e.g., call or video conference), third-party identity services (e.g., Veriff, Plaid, DigiLocker), or government-issued ID documents. This was a deliberate choice to rely on general approaches to verifying customer identity that do not rely directly on the customer’s relationship to the life sciences, inspired by approaches described in NIST’s SP 800–63 Digital Identity Guidelines (National Institute of Standards and Technology, 2025), as highlighted within Crawford et al. (2024). These approaches, already used by commercial entities for verifying financial, social media, and other accounts, have not often been linked to nucleic acid synthesis screening, but could be particularly helpful when a customer’s institution is less well-established or if other ambiguities arise about their legitimacy as part of the life sciences community.

Providing options for verifying customer identity with “some” or “enhanced” due diligence, rather than recommending a single approach, is a design choice that reflects a trade-off between providing streamlined, standard instructions and ensuring that legitimate customers in diverse countries and institutional settings are able to access sequences. Developing standardized approaches that account for different institutional structures and documentation practices across countries would help ensure that legitimate international customers are not disadvantaged by screening processes designed around specific national contexts.

### 2.3 Customer legitimacy

For orders that contain SOCs, current policies and guidance recommend verifying the legitimacy of the customer. As shown in Table 1, guidance varies on what indicators of legitimacy are requested, and reliable indicators of legitimacy are likely to differ across countries and contexts. We have heard the phrase “customer legitimacy” itself questioned by synthesis providers piloting early versions of the IBBIS forms, who worry their customers will be offended by the suggestion that their legitimacy is not self-evident. However, the language of “legitimacy” is used by all four policies and so we continue using it and it remains in the IBBIS guidance. One of the most common indicators of customer legitimacy, considered by every synthesis provider we consulted and recommended by every policy in Table 1, is the customer’s institutional affiliation and institutional legitimacy.

The customer’s institutional affiliation is requested as part of the New Customer Form, and the SOC Order Form calls for confirming that the customer’s email and shipping address match the institution, or that the customer has provided a reasonable explanation for why they do not. The SOC Order Form requests that the customer provide additional documentation to show one or more of the following indicators of individual legitimacy: research history (e.g., ORCID, links to publications or conference participation), institutional affiliation (e.g., profile on institutional website), project documentation (e.g., grants, press releases), or government-issued approvals (e.g., licenses to work with regulated pathogens, export control forms, or business licenses).

The decision guidance calls for verifying that documentation is recent, consistent with other information provided in the form, and aligned with the specific SOCs ordered. This reflects guidance in ISO 20688–2 that a provider should “verify that the information obtained … is consistent with the customer’s activities” (International Organization for Standardization, 2024, p. 7) and in the IGSC Harmonized Screening Protocol to verify the information is both “self-consistent (in that the Customer appears to have sufficient expertise to understand the planned use for ordered sequences) and … consistent with the nature of the ordered sequence(s)” (International Gene Synthesis Consortium, 2024, p. 11).

Customers with a formal institutional approval process may use that process as an indicator of their legitimacy. The SOC Order Form requests contact information for a “biosafety officer, biorisk manager, biosafety professional, or other professional responsible for overseeing biorisk for the project” who signs the form to indicate institutional approval. However, many non-academic institutions do not have a formal institutional approval process, and practices such as research registration and review by an institutional biosafety committee are not standard in every country. An early draft of the SOC Order Form requested institutional approval sign-off for any “established life sciences institution [with] ongoing, publicly documented, and well-established activities related to the life sciences,” and requested alternative indicators for any “new or less-documented life sciences institution,” but we received feedback that there are well-established, highly-reputable research labs (including some funded by the Institut Pasteur and CEPI) without a formal institutional approval process. If the institution is unfamiliar, or when the end user cannot provide formal institutional approval, the SOC Order Form recommends verifying at least two of the following indicators of legitimacy: that the institution is an established legal entity; that it provides biosafety oversight or training; that it has a life sciences mission that it is part of the scientific community; or that it has obtained regulatory approval or official certification for work with life sciences materials.

Legitimate life sciences institutions range from well-established research universities to start-up companies to public health organizations to community laboratories. Customer screening systems must create clear pathways for researchers to demonstrate their legitimacy regardless of their institutional setting or geographic location.

### 2.4 Legitimate use

When a customer orders a sequence of concern, current guidance recommends not only verifying that customer legitimacy in general, but that the customer has a legitimate use for the specific SOCs ordered. Established guidance and policies generally require that nucleic acid providers evaluate written descriptions of how the order will be used. Customers are often unwilling to provide sufficient detail—for example, nucleic acid providers have reported that customers often write, simply, “research purposes.”^1^ Even detailed descriptions can be difficult to interpret; in one IBBIS-hosted workshop, a participant with a PhD in biomedical engineering, asked to evaluate biosecurity risks from sequences flagged as derived from the organisms *Xanthomonas citri*, *Plasmodium falciparum* and *Escherichia coli* O157 (among several others), said he had “new appreciation for the other side” and the difficult job of screening; experts in one of these pathogens may not know much about the biosecurity risks or legitimate uses associated with the others.

The SOC Order Form requests a written description of intended use, consistent with other guidance, but IBBIS designed the form to supplement this with other indicators rather than relying on customer descriptions.

If a customer’s work has been approved after institutional review by a “biosafety officer, biorisk manager, biosafety professional, or other professional responsible for overseeing biorisk for the project,” the synthesis provider need not make an independent assessment of the planned use. Streamlined methods for communicating institutional approval would be useful; the IBBIS forms allow an institutional contact to sign, indicating approval, but it is not always easy for nucleic acid providers to make contact with the relevant institutional biosafety officials, especially in the face of impatient customers and international variance in oversight practices. Even detailed approvals do not list every individual that might contribute to a project, nor every sequence that an end user might work with.

As discussed in the previous section, much legitimate science is done in environments without an institutional approval process. If the customer is not subject to institutional oversight and approval, the SOC Order Form requests other evidence that an end user has a reason to order particular sequences, such as relevant past publications, ongoing collaborations, grants, business plans, media stories, or industry memberships. However, this process is laborious and it can be difficult to connect such diverse evidence to specific SOCs that a researcher might legitimately order.

Another useful indicator of legitimacy is approval under local licensing requirements or export controls. The decision guidance recommends that providers check that the end user and institution have appropriate permits or registrations. However, providers may not be familiar with the details of local licensing requirements in each jurisdiction they serve, and are not typically obligated to ensure that their international customers have local regulatory approvals. By contrast, export licenses are granted directly to the provider rather than the customer, though customers often provide information as part of the licensing process. The SOC Order Form asks customers to identify potential restrictions but places responsibility on providers to resolve requirements, acknowledging that customers may not fully understand export control obligations.

### 2.5 Supply chain screening

Customers of synthetic nucleic acids are often envisioned as individuals who design and place an order for a sequence from a large, commercial provider and then receive the synthesized nucleic acids directly from that provider. However, recent guidance and policies have acknowledged the complexity and diversity of provider-customer relationships by providing definitions for “customer,” “principal user,” and “end user.” They have also expanded the guidance for “providers” to include responsibilities for non-traditional providers (such as the U.S. policy’s explicit inclusion of academic core facilities, cloud labs, and contract research organizations (Office of Science and Technology Policy, 2024, p. 7)) as well as third-party resellers of synthetic nucleic acids.

Non-traditional or third-party vendors and their business models are very diverse (Table 3), and customer screening in these contexts may require more specialized guidance. Some non-traditional synthesis providers have lower order volumes and higher interaction with their customers than current guidance seems to assume; for providers such as core facilities and specialist CROs, much of the information relevant to customer legitimacy and intended use accumulates through normal business interactions such as contract negotiation. By contrast, when a third-party vendor places an order, the nucleic acid provider who synthesizes the sequences often has limited interaction with, and sometimes is unaware of, the ultimate end user. The third-party vendor is in a better position to verify the end user’s legitimacy, the legitimacy of their institution, and legitimate use. Given these multiple layers of responsibility, it can be difficult for these different types of providers to understand their roles.

**TABLE 3:** Examples of diversity among non-traditional and third-party vendors.

Example	Order volume	Customer interaction
A country-specific reseller of synthetic nucleic acids (e.g., FastBio in Brazil, Premas Life Sciences in India, or Takapouzist Co. in Iran). The company accepts customer orders, requests the orders from an international partner, then handles all the logistics of delivering from the partner to their country	High	Low
A company specialized in creation of custom viral vectors. The company receives designs from users, orders synthetic nucleic acids from a traditional nucleic acid provider, clones the custom design into one of their proprietary vectors, then ships the assembly to their own customers	Moderate	Moderate
An academic core facility which does not have synthesis equipment in-house, but performs complex DNA assemblies and regularly orders synthetic nucleic acids to be assembled. No researchers from outside the academic institution have access to these services	Low	High

To reflect this diversity, the IBBIS New Customer Form asks whether customers are the intended end user of the synthetic nucleic acids. If not, the form asks whether the customer is at the same institution as the end user or a third-party vendor. For customers such as institutional procurement officers, the form requests contact information for an additional person at the institution, allowing some due diligence for institution-level legitimacy. If the customer is a third-party vendor, the form requests that the vendor provide documentation of institutional legitimacy similar to what is requested on the SOC Order Form, and the decision guidance suggests conducting some due diligence to verify the legitimacy of the third-party vendor as an institution.

The IBBIS SOC Order Form also requests additional information if the customer is not the end user, including requesting that the customer obtain information from the end user to determine legitimacy, institutional affiliation, and intended use of the product. The form includes a dedicated section where third-party vendors can attest that they will conduct sequence and customer screening, following the recommendation from the IGSC Harmonized Screening Protocol to only sell to distributors or other resellers if “those companies agree to identify the end-user receiving the products and demonstrate their compliance with every requirement otherwise applicable to that end-user” (International Gene Synthesis Consortium, 2024, p. 15) and recommendations in the US framework that third party legitimacy should also be assessed. However, these methods currently rely on proactive disclosure by third-party vendors that they are not the end user and are scant on procedural details specific to supply chain screening.

## 3 Recommendations to advance customer screening

It was possible to create forms and decision guidance that reflect the emerging consensus on customer screening, but this exercise also highlighted persistent ambiguities that must be resolved to transform broad best practices into clear standards. The recommendations in this section would reduce ambiguity, streamline procedures for legitimate customers, and support best practices for customer screening.

We have focused below on recommendations that we believe could support implementation of customer screening across the industry without requiring significant formalization or policy action. However, the broad adoption of standards will require coordination among the synthesis industry, biosecurity researchers, and policymakers, and we expect it to advance through multiple complementary processes: voluntary adoption by industry consortia such as the IGSC and DDSA (SNIA DNA Data Storage Alliance, 2025); implementation guidance for formal international standards, such as guidance for ISO 20688–2 currently under development by an IBBIS-led consortium; and government-led certification or licensing, including those anticipated under revisions to the US framework and UK guidance. An example roadmap for moving synthesis screening from voluntary attestation to enforceable certification has been described elsewhere (Beal and Alexanian, 2025); customer screening fits into this trajectory, and we believe the recommendations below would support early standards development whether through industry, international, or regulatory avenues.

### 3.1 Establish minimal globally-relevant baseline for customer screening

Recent national and international policies have encouraged adoption of synthesis screening through voluntary guidance and self-attestation. Eventually, however, this adoption should be strengthened by adoption of enforceable standards and real compliance requirements.

Customer screening standards are neither established nor harmonized enough that providers can be certified as compliant at customer screening. However, similar to recent definitions of a baseline set of sequences of concern (Laird et al., 2025), which all responsible providers agree should be flagged, it should be possible to define a baseline set of customer attributes which all providers agree should merit some form of additional customer screening. A baseline could include, for example, follow-up screening for any customers that attempt to be anonymous, that request shipping to a residential address, or that have a name matching the UN sanctions list. It could also include acknowledgment that, when applicable, export controls and national licensing requirements should be followed.

A globally-relevant baseline should specify *what* providers must verify, while accepting different evidence based on the local context. In some jurisdictions, all customers working with recombinant DNA can readily produce a government-issued license; in others, no such national licensing exists. The focus on minimal verification outcomes should allow the creation of a customer screening baseline that could be implemented in every region with significant synthesis activity.

The benefit of a minimal baseline is to distinguish between providers that conduct any meaningful screening and those that do not, enabling enforceable standards in advance of perfect agreement on edge cases. In the case of sequences of concern, most responsible providers flag far more sequences than the baseline for which an unambiguous consensus can be achieved. Customer screening practices can be refined over time and implementation can be streamlined, allowing enforceable standards to eventually expand beyond the minimum.

### 3.2 Clarify ambiguities through practical case studies

The minimal baseline proposed above is intentionally limited to areas of existing consensus. However, ambiguous customer scenarios continue to challenge synthesis providers, and a shared understanding of best practices should be built to expand consensus.

Already, case studies of different customers, paired with specific orders, have been developed to explore screening in practice. IBBIS has developed an order screening game with a dozen customer personas (Alexanian, 2024) that have been used by synthesis providers to train customer service representatives, as well as during workshops for policymakers. In addition, six screening case studies were published in the Bulletin of the Atomic Scientists (Batalis and Venkatram, 2025), and the Engineering Biology Research Council is developing customer profiles to use in testing provider screening systems (Engineering Biology Research Consortium, 2025a).

Additional case studies would be particularly helpful to address global verification of customer legitimacy and effective supply chain screening. First, case studies of legitimate customers with broad geographical and sectoral representation (including those proposed in Section 3.4) would support legitimacy verification in diverse countries and settings, preserving access as customer screening becomes more standard and stringent. Second, real-world examples of non-traditional and third-party vendors (see Table 3), with details on how orders map to end users, what services they provide, and their current customer screening practices, would reveal where current guidance remains ambiguous. For example, operationalizing the expectation in the IGSC protocol that third-party vendors ”demonstrate their compliance with every requirement otherwise applicable to that end-user” (International Gene Synthesis Consortium, 2024, p. 15) will be easier with realistic case studies.

### 3.3 Encourage automated identity verification with clear guidance

Identity verification remains labor-intensive and inconsistent across providers, but established tools and standards from other industries could streamline these processes, as could some additional resources built specifically for the synthesis industry. Automated identity verification could be encouraged through.

- Best practices for customer screening to make use of existing software for digital identity verification (such as Veriff, Plaid, CLEAR, Persona, or DigiLocker) and sanctions screening (such as LexisNexis and LSEG World-Check).
- Transferable identity verification for life sciences customers, users, or community members. For example, identity verification could be connected to Open Researcher and Contributor IDs (ORCID), which are commonly used in the life sciences community, or Global Alliance for Genomics and Health (GA4GH) data passports.
- Best practices for establishing the legal status of smaller or newer life sciences institutions, including lessons from the financial sector and the use of persistent identifiers such as Research Organization Registry (ROR) identifiers.

### 3.4 Leverage existing approvals to demonstrate customer legitimacy

Rather than each provider independently assessing customer legitimacy, screening systems should build on existing institutional oversight and government approvals where they exist. These mechanisms include institutional biosafety committee reviews; national licenses for work with recombinant DNA or specified pathogens; and approvals from science funders or sector-specific oversight bodies. Recent proposals, such as the UK SCAN system for automated screening of access to biological tools (Moulange et al., 2026), may also create new approvals that can be used to indicate legitimacy. Available approvals vary based on local regulatory context and the specific sequences ordered; the recommendations below focus on creating methods and reference datasets that would make it more straightforward to integrate approvals into customer screening.

- Streamlined methods for communicating approvals from institutions, science funders, or other third parties for end users to work with specific biological agents, SOCs, or biosafety containment levels. One pilot project in this area is SecureDNA’s Exemption Certification System (Baum et al., 2026) that integrates biosafety officer approvals with sequence screening.
- Methods to automatically collate public information about whether an individual has research history with specific biological agents or SOCs. Recent work has evaluated AI-assisted approaches for this type of customer verification, finding that frontier models with bibliographic and sanctions APIs can match expert human accuracy at roughly one-tenth the cost (Acelas et al., 2026).
- A reference dataset indicating whether customers or institutions in particular countries are required to have institutional biosafety approvals or government-issued permits (such as the licenses issued by the UK SAPO and US FSAP programs) to conduct life sciences work or possess SOCs. Some of this work should be completed as part of the IBBIS Global DNA Synthesis Map (International Biosecurity and Biosafety Initiative for Science IBBIS, 2025), and an expanded reference dataset would allow providers to know which approvals they should expect in each jurisdiction.

### 3.5 Integrate sequence and customer screening

Tiered customer screening should include appropriate due diligence at additional tiers of risk beyond “all orders” and “orders including SOCs.” IBBIS has developed a draft version of such guidance (see Supplementary Material), but additional refinement and feedback from the broader community is needed. Further work in this space will require closer integration of sequence and customer screening methods.

- Standardizing sequence screening outputs (see Table 2) to reflect shared tiers of risk, including standard ways to describe pathogen-related sequences that do not pose significant biosafety or biosecurity risks (e.g., metabolic or housekeeping genes).
- Cross-referencing of existing tiered identity verification guidelines, such as NIST’s SP 800–63 (National Institute of Standards and Technology, 2025), to nucleic acid orders that pose different levels of biosafety or biosecurity risk.
- Government-issued guidance on which specific sequences (rather than organisms or broad categories like sequences that “endow or enhance pathogenicity”) require export licenses or government-issued permits. For example, the April 2023 update to France’s microorganisms and toxins list specifies controls based on sequence length and named genes of concern (Ministère de la Santé et de la Prévention, 2023).

## 4 Discussion

The consensus on customer screening practices emerging from recent policies provides a solid foundation, but does not resolve implementation challenges across the nucleic acid synthesis industry. For each area of emerging consensus, we outline specific tools and resources that would advance best practices. The IBBIS customer screening forms and decision guidance demonstrate one approach to translating policy consensus into practical tools, but significant opportunities remain to enable synthesis providers to conduct customer screening more efficiently, consistently, and confidently.

Supply chain screening remains the least operationalized across the five areas of emerging consensus. Third-party vendors and non-traditional providers are acknowledged, but best practices for end user verification across complex supply chains are not specified, nor are less formalized approaches for providers with high levels of customer interaction. Additional investigation into the structure of existing supply chains, and the customer interactions they currently support, is needed before standards for supply chain screening can be enforced.

Many of our suggestions point to a future in which customer legitimacy is established more proactively. The burden of verifying customer identity could be reduced by associating existing customer identifiers, such as persistent digital identifiers (e.g., ORCID) or institutional email addresses, with a higher level of identity verification. More ambitiously, a pre-approval mechanism that acts as a trustworthy and transferable indication of customer legitimacy and legitimate use has been identified as a way to reduce the screening burden placed on individual synthesis providers (Baum et al., 2026; Carter, 2024; Rose et al., 2024; Crawford et al., 2024; Moulange et al., 2026). This type of pre-approval could draw on existing institutional or government oversight or be based on new third-party certifiers.

The future of customer screening likely also includes a greater participation of life sciences institutions. Universities, funding agencies, and providers of life sciences products other than synthetic nucleic acids could take on larger roles in verifying customer identity, customer legitimacy, and legitimate use, reducing the burden of screening for any specific synthesis provider or sequence order. A broader support system for customer screening will reduce biosecurity risks across the life sciences, increase confidence in the legitimacy of the scientific enterprise, and help build public trust in scientific institutions.

Although recent policy development for customer screening has focused on nucleic acid synthesis screening, the verification standards, digital identity tools, and other resources discussed in this paper create precedents for securing emerging biotechnologies more broadly. The UK guidance already outlines screening obligations for sales of proprietary and sole-use synthesis reagents, and we expect customer screening will expand across life sciences supply chains, beyond providers of synthetic nucleic acids. Establishing streamlined, rigorous, and transparent customer screening will allow biosecurity concerns to be addressed while preserving the openness and international collaboration that enables modern life sciences research.

## Footnotes

1 Contributions to a workshop under the Chatham House Rule from an industry participant. Workshop was hosted by the Engineering Biology Research Consortium, September 10–11, 2024

## Supplementary material

Supplementary file 1 Appendix A: Tiered Customer Screening Practices in the Life Sciences, containing a framework for five tiers of customer screening with corresponding verification requirements (Table∼S1).

Supplementary file 2 Appendix B: Customer Screening Guidance in National and Internation Policies: 2024 Reference, containing a compilation of guidance on customer screening from each of the four policies references in table [consensus].
