# Supplementary material for: Implementing emerging customer screening standards for nucleic acid synthesis
Source: Front Bioeng Biotechnol. 2026 Jul 29;14:1819810. doi: 10.3389/fbioe.2026.1819810 (PMC13462936; doi:10.3389/fbioe.2026.1819810)
Supplement: Supplementary file 1 [file Supplementaryfile1.pdf]

frontiers

Supplementary Material

1 APPENDIX A: TIERED CUSTOMER SCREENING PRACTICES IN THE LIFE SCIENCES

Many life sciences products and services pose very little biosafety and biosecurity risk, while others can pose significant risks. Providers of life sciences products and services should thoughtfully determine what level of customer verification their products require.

For customers who are the end users of the products they order, we establish the following tiers:

- **Tier 0:** Establishing a commercial relationship (not life-sciences-specific).
- **Tier 1**: Customer onboarding (equivalent to New Customer Form).
- **Tier 2**: Products that pose low levels of biosecurity risk (second approver, end use).
- **Tier 3**: Products that pose moderate levels of biosecurity risk (institutional legitimacy).
- **Tier 4**: Products that pose higher levels of biosecurity risk (additional individual and institutional legitimacy, equivalent to SOC Order Form).

We propose customer screening practices for each level in Table S1. The highest tiers are intended to gather information to establish that a customer and their institution can be trusted to use life sciences products or services with the highest biosafety and biosecurity risks.

Table S1. Tiered Customer Screening Practices

Tier	Customer Identity	Customer part of life sciences community	Institutional legitimacy	Institutional responsibility or approval
**Tier 0**	Check government watchlists. Automated check of email address or phone number for successful contact.			
**Tier 1**	Check that email address, phone, shipping address are consistent with institution. Check government watchlists.	Request institutional affiliation.		
**Tier 2**				Request information about how the ordered products will be used. Request contact information for another person at the institution, such as a biosafety officer. Check email address or phone number for successful contact with the second person.
**Tier 3**			Request documentation to establish that the institution is legitimate.	
**Tier 4**	Call, conduct a video conference, or otherwise establish real-time contact to verify identity. **OR** Verify identification using an established third-party service (e.g. Veriff, Plaid, CLEAR, Persona, or DigiLocker).	Request documentation to establish that the customer is a member of the life sciences community.	Request documentation to establish that the institution is a part of the life sciences community.	Request signed approval from institutional biorisk management for ordered products.
**Additional verification measures for high-risk products or services**	Request a copy of a government-issued ID or other identifying documents. Visit institution.			Call, conduct a video conference, or otherwise contact biosafety officer (or other listed contact) to verify identity, affiliation, and approval of project. Visit customer at their institution.
