# Supplementary material for: Implementing emerging customer screening standards for nucleic acid synthesis
Source: Front Bioeng Biotechnol. 2026 Jul 29;14:1819810. doi: 10.3389/fbioe.2026.1819810 (PMC13462936; doi:10.3389/fbioe.2026.1819810)
Supplement: Supplementary file 2 [file Supplementaryfile2.pdf]

IBBIS

International Biosecurity and Biosafety Initiative for Science

CUSTOMER SCREENING GUIDANCE IN NATIONAL AND INTERNATIONAL POLICIES

2024 REFERENCE

Tessa Alexanian, Sarah R. Carter | June 2025

This document collects customer screening guidance from 4 policies issued in 2024:

1. **US Framework for Nucleic Acid Synthesis Screening**
April 2024, *United States Office of Science and Technology Policy*.
https://aspr.hhs.gov/S3/Pages/OSTP-Framework-for-Nucleic-Acid-Synthesis-Screening.aspx
2. **UK screening guidance on synthetic nucleic acids for users and providers.** October 2024. *United Kingdom Department for Science, Innovation, and Technology*. https://www.gov.uk/government/publications/uk-screening-guidance-on-synthetic-nucleic-acids/uk-screening-guidance-on-synthetic-nucleic-acids-for-users-and-providers
3. **IGSC Harmonized Screening Protocol© v3.0.** September 2024. *International Gene Synthesis Consortium.*
https://genesynthesisconsortium.org/wp-content/uploads/IGSC-Harmonized-Screening-Protocol-v3.0-1.pdf
4. **ISO 20688-2:2024**. Biotechnology—Nucleic acid synthesis, Part 2: Requirements for the production and quality control of synthesized gene fragments, genes, and genomes. March 2024. *International Organization for Standardization*. https://www.iso.org/standard/75852.html

US Framework for Nucleic Acid Synthesis Screening

3) Screen customers who submit purchase orders of synthetic nucleic acids with SOCs, and of benchtop nucleic acid synthesis equipment, to verify legitimacy

To adhere to this screening framework, Providers and Manufacturers should assess customer risk by following the 2023 HHS Guidance and industry standard “know your customer” practices. As noted in the 2023 HHS Framework, this includes assessing customer identity for all orders, such as point-of-contact name, institution, address, and contact information, and verifying customer legitimacy for orders containing SOCs. Notwithstanding the previous sentence, the Provider may still be adherent to this Framework without verifying customer legitimacy for exempted SOCs as described in the preceding section.

To adhere to this screening framework, Providers should develop and implement a process to assess the legitimacy of orders that have been identified by the sequence screening protocol as containing a SOC. The legitimacy of an order is determined by verifying the legitimacy of both the individual customer placing the order and their institution.

To adhere to this screening framework, Providers and Manufacturers should confirm the legitimacy of the individual customer by ensuring that the person (or customer) placing an order has no red flags *(As defined in the 2023 HHS Guidance including its Companion Guide)*, is affiliated with a legitimate institution, and has a legitimate need for using synthetic nucleic acids.

To adhere to this screening framework, Providers and Manufacturers should confirm the legitimacy of the institution by verifying its legal standing and that it has a life sciences-oriented mission and purpose, or uses synthetic nucleic acids for other relevant applications *(Such as DNA storage or other emerging applications of synthetic biology)*, and ensuring there are no red flags *(As defined in the 2023 HHS Guidance including its Companion Guide)*.

To adhere to this screening framework, at a minimum, Providers should include a field or mechanism in their ordering system where customers can self-identify that an order contains a SOC. When an order does contain a SOC, Providers should also include a mechanism for customers to provide information that will be useful for verifying the customer’s legitimacy *(While other types of information may also be useful for verifying customers’ legitimacy—as determined by individual Providers and Manufacturers—a mechanism for providing at least the following information should be included: proposed end use of the order, institutional or corporate affiliation (if applicable), the name of a biosafety officer (if available), documentation of internal review and approval of the research, evidence provided by the recipient’s Responsible Official that the recipient is registered with the Federal Select Agent Program (FSAP) or Statement by Ultimate Consignee and Purchaser (i.e., a completed BIS-711 form, if applicable), other evidence of a legitimate research or training program (e.g., publication history, researcher persistent identifiers such as ORCID, business licenses, grant numbers, research plan) or other legitimate use (e.g., diagnostic test development or manufacture)*. While all these types of information are not likely needed to verify individual instances of legitimacy, the opportunity to provide all of them should be included in ordering materials.). In addition, Providers should ask customers if they are the end user for the SOCs or

if the order will be passed along to a third party(s), in which case the legitimacy of the third party(s) should also be assessed.

To adhere to this screening framework, Manufacturers should develop and implement a process to assess the legitimacy of orders for their equipment, such as through verified user accounts.

As stated above, the legitimacy of an order is determined by verifying the legitimacy of both the individual customer placing the order and their institution—which should include verification that the institution will cooperate in ensuring that benchtop synthesizers are only accessed by legitimate end users.

Manufacturers should implement mechanisms to track legitimate use of their equipment, including when it is potentially transferred to a new user during the lifecycle of these equipment. Ensuring that benchtop nucleic acid synthesis equipment is oconnly sold to legitimate customers and end users in this context also includes ensuring that proprietary and sole-use reagents for their benchtop synthesizers *(Reagents that can only be obtained from the manufacturer for their equipment and do not have common applications other than the operation of their equipment.)* are only sold to legitimate customers and end users, even if they were not screened for legitimacy when initially obtaining their benchtop nucleic acid synthesizer (e.g., if they acquired their equipment prior to the time this framework comes into effect).

Definitions

Term	Definition
Customer	The individual or entity (such as an institution) that orders or requests synthetic nucleic acids from a Provider, or that purchases nucleic acid synthesis equipment from a Manufacturer.
Provider	An entity that synthesizes and distributes synthetic nucleic acids. Providers may provide nucleic acids to a customer or third-party vendor. A Provider is understood to be synthesizing and distributing nucleic acids as a transactional service, rather than as a research scientist collaborating with a colleague.
Manufacturer	An entity that produces and distributes benchtop equipment for synthesizing nucleic acids. Manufacturers may provide equipment to a customer or third-party vendor.
Third-party vendor	An entity that orders synthetic nucleic acids from Providers and distributes them or their constructs, with or without reformulation. Also, an entity that orders benchtop equipment for synthesizing nucleic acids from Manufacturers and distributes them.
Sequence of Concern (SOC)	At the time of this framework's issuance, a nucleotide sequence or its corresponding amino acid sequence that is a Best Match to a sequence of federally regulated agents (i.e., the Biological Select Agents and Toxins List (BSAT), or the Commerce Control List (CCL)), except when the sequence is also found in an unregulated organism or toxin. As of and after October 13, 2026, this definition will include sequences known to

	contribute to pathogenicity or toxicity, even when not derived from or encoding regulated biological agents.
Benchtop nucleic acid synthesis equipment	Benchtop nucleic acid synthesis equipment sold by Manufacturers that is intended to be used to synthesize nucleic acids for use within a research laboratory or within an institution. While this nucleic acid synthesis equipment may not be small enough to be placed on a benchtop (e.g., it sits on the laboratory floor), it is still considered benchtop equipment if it is sold with the intent that it will be used by researchers individually or in a core facility in an institution.
Verifying legitimacy	Review of information that would allow Providers and Manufacturers to authenticate the recipient of synthetic nucleic acids containing SOCs orb enchtop nucleic acid synthesis equipment as a legitimate member of the scientific community.

UK screening guidance on synthetic nucleic acids for users and providers

Customer Screening

Providers and third-party vendors

- Providers and third-party vendors should know and document who they are distributing to.
- Upon receiving an order for synthetic nucleic acids, providers and third-party vendors should verify the identity of the customer see customer screening methodology.
- Providers and third-party vendors should implement adequate security and cybersecurity measures to protect the intellectual property and identity of customer.
- Providers should retain customer screening information and refer to it for repeat orders. At least every 18 months the provider should ask the customer to update their information.

Benchtop manufacturers

- Only distribute equipment capable of synthesising nucleic acids containing SOCs to users whose:
  - legitimacy has been verified, and
  - implemented mechanisms to ensure that the devices are only operated by legitimate users.
- Manufacturers whose benchtop nucleic acid synthesisers require the use of proprietary and sole-use reagents (i.e., reagents that can only be obtained from the manufacturer of their devices and do not have common applications other than the operation of their devices) should screen users purchasing those reagents to verify their legitimacy. This should take place even when they were not screened when obtaining their nucleic acid synthesiser (i.e., when they acquired their device prior to the issuance of this gene synthesis guidance).
- Manufacturers should implement mechanisms to track legitimate use of their equipment, including when it is potentially transferred to a new user during the lifecycle of the equipment.
- Manufacturers are encouraged to have a closed loop system in which operation of their devices relies upon obtaining reagents only available from the manufacturers (who establish the legitimacy of users whenever they obtain these reagents).

Users

- Users should be forthcoming in providing data on their identity and legitimacy.

- Users should notify the transfer of their benchtop synthesisers to new users.

Customer Screening: methodology

Verification of customer legitimacy should include collection and review of the following pieces of information:

- name and address of customer
- if the customer is not an individual, the name of an individual who is authorised by the customer to acquire synthetic DNA
- a statement of the nature of the user's purpose

In addition to the above, one or more piece of the following information should be requested:

- name of the institutional biosafety officer
- publication history
- open researcher and contributor identifier

VAT registration number [if UK based], ie the number allocated by the Commissioners for His Majesty's Revenue and Customs to a person registered under the Value Added Tax Act 1994

The same criteria should be applied to verify the legitimacy of any user of synthetic nucleic acid or benchtop nucleic acid synthesis equipment.

Providers should retain customer screening information and refer to it for repeat orders. At least every 18 months the provider should ask the customer to update their information.

Screening order legitimacy

If a provider deems the transaction as 'suspicious', they should conduct follow-up screening, focusing on the legitimacy of use. Providers should contact users to understand the purpose and end-use of the order.

Providers

- If the order contains a SOC listed in Schedule 5 of the ATCSA, the Provider must ask the user to provide proof that the user has notified the Home Office under Part 7 of the ATCSA and undertake follow-up screening to determine the legitimacy and purpose of the order
- If the order contains SOC's that are derived from an animal pathogen listed in in Group 3 or 4 in Schedule 1 of SAPO, that could produce that pathogen when introduced into a biological system in which the nucleic acid is capable of replicating, providers must ask the user to provide proof that they have obtained a licence through HSE
- If the order contains sequences they deem to be SOC's, but are not listed in existing legislation, the provider should undertake follow-up screening to determine the legitimacy and purpose of the order

Users

- Users who know that their synthetic nucleic acid order contains SOCs should pre-emptively provide information that will assist the provider or third-party vendor in verifying the legitimacy of their use.
- When requested by providers, users should be forthcoming in providing information on the purpose of the end-use of their order.

If providers' concerns are not alleviated through follow-up conversations, they should not fulfil the order, and should contact biological.reporting@met.police.uk.

Definitions

Term	Definition
Customer user	The individual or entity (such as an institution) that orders or requests synthetic nucleic acids from a provider, or that purchases synthesis equipment from a manufacturer.
Provider	Any organisation that synthesises and distributes synthetic nucleic acids to a user.
Manufacturer	An organisation that produces and distributes benchtop equipment for synthesizing nucleic acids. Manufacturers may provide equipment to users or third-party vendors.
Third-party vendor	An entity that orders synthetic nucleic acids from providers and sells them or their constructs, with or without reformulation, or re-sells benchtop equipment for synthesising nucleic acids.
Principal user	The individual who originates an order or request for synthetic nucleic acids or synthesises nucleic acids and oversees the use of ordered or synthesised nucleic acids in the laboratory. The principal user may also be the end user.
End user	The individual who uses the synthetic nucleic acids.
Synthetic nucleic acid	Molecules that have been synthetically constructed outside of living cells, that are constructed by joining nucleic acid molecules. The definition includes sequences of any length.
Sequence of Concern (SOC)	A nucleotide sequence that is known to contribute to pathogenicity, virulence, or toxicity. SOCs are sequences that are a best match to current and future iterations of regulated agents, except when the sequence is also found in an unregulated organism or toxin.<br>Key regulation includes:<br>- Schedule 5 of the Anti-terrorism, Crime and Security Act 2001<br>- Group 3 and 4 in Schedule 1 of the Special Animal Pathogens Order (SAPO)

	- Hazard group 3 and 4 in the Approved List of biological agents under the Schedule 3 of Control of Substances Hazardous to Health Regulations 2002 (COSHH)<br><br>- Human and animal pathogens and toxins for export controls in the UK Consolidated Strategic Export Controls List
Benchtop nucleic acid synthesis equipment	Benchtop nucleic acid synthesis equipment sold by Manufacturers that is intended to be used to synthesize nucleic acids for use within a research laboratory or within an institution. While this nucleic acid synthesis equipment may not be small enough to be placed on a benchtop (e.g., it sits on the laboratory floor), it is still considered benchtop equipment if it is sold with the intent that it will be used by researchers individually or in a core facility in an institution.

IGSC Harmonized Screening Protocol v3.0

Assessing Risk and Legitimate Use

The U.S. Guidance indicates that Providers should ensure that Customers have a 'legitimate, bona fide and peaceful purpose' for purchased synthetic nucleic acids that contain Sequences of Concern. There is, however, no agreed-upon framework (within any country, much less internationally) against which Providers could measure a Customer's stated use to determine its legitimacy. There is also no framework available for consistently categorizing specific biological risk posed by a given construct.

Given this lack of formal frameworks for understanding and recording risk, Providers should ensure that in discussions with Customers, the stated intended use for ordered Sequence of Concern nucleic acids is a) self-consistent (in that the Customer appears to have sufficient expertise to understand the planned use for ordered sequences) and b) consistent with the nature of the ordered sequence(s).

This series of judgments by the Provider generally requires significant scientific expertise on the part of the individual representing the Provider in these conversations. It is common for these individuals to have doctoral degrees and significant experimental experience. It is additionally valuable for these individuals to have a background or training in the history of biological weapons and an understanding of agents and toxins traditionally considered weaponizable.

Such a background will maximize the potential for Providers to detect potential misuse of a Sequence of Concern.

Individuals with insufficient expertise will be less likely to detect when the provided intended use makes little sense or is contradicted by the nature of the ordered sequence(s).

Customer Screening Protocol

Step	Description
1.1	IGSC members require identification data from all potential Customers for synthetic nucleic acids, including at a minimum a shipping address, institution name, country, telephone number, and email address. We do not ship to residential addresses or to PO Boxes.
1.2	Potential Customers are subject to restricted party screening against any relevant lists of sanctions or denied party lists maintained by countries relevant to each Provider's base of operations. Relevant lists include the U.S. OFAC's SDN List, the U.S. Department of State Debarred List, and U.S. BIS's Denied Persons, Entity, and Unverified lists, or the German<br><br>HADDEX list, and/or any other list required by applicable national regulations.

1.3	IGSC members require additional Customer screening before accepting orders for nucleic acid sequences from regulated pathogens or toxins. IGSC members supply nucleic acid sequences from regulated pathogens and toxins only to researchers in bona fide government laboratories, universities, non-profit research institutions, or industrial laboratories demonstrably engaged in legitimate research. Customers ordering significant portions of the genomes from regulated pathogens or toxins must provide a written description of the intended use of the synthetic product. IGSC companies should verify independently a) the identity of the potential Customer and purchasing organization, and b) that the described use is consistent with the activities of the purchasing organization.
1.4	IGSC members use the current recommendations from the U.S. CDC and/or the Department of Agriculture and/or the European Commission to determine which nucleic acid sequences are Select Agents as recombinant nucleic acid fragments. We supply such sequences only if the supplier and the Customer are able to comply with all Select Agent regulations applicable to that sequence.
1.5	In general, IGSC members only sell DNA or fragments of regulated pathogens to bona fide end-users. We do not sell or ship such material to distributors or other resellers, unless those companies agree to identify the end-user receiving the products and demonstrate their compliance with every requirement otherwise applicable to that end-user, or are themselves members of the IGSC that follow the Harmonized Screening Protocol.

Definitions

Term	Definition
Customer	The individual or entity (such as an institution) that orders or requests synthetic nucleic acids from a Provider, or that purchases synthesis equipment from a Manufacturer.
Provider	The entity that synthesizes and distributes synthetic nucleic acids to a Customer. A Provider is understood to be synthesizing nucleic acids as a transactional service, rather than a research scientist collaborating with a colleague.
Manufacturer	An entity that produces and sells benchtop equipment for synthesizing nucleic acids. Manufacturers may provide equipment to individuals, entities, Principal Users, or Third-Party Vendors.
Third-party vendor	An entity that orders synthetic nucleic acids from Providers and sells them or their constructs, with or without reformulation, or resells benchtop equipment for synthesizing nucleic acids.
Principal user	The individual who originates an order or request for synthetic nucleic acids or synthesizes nucleic acids and oversees the use of ordered or

	synthesized nucleic acids in the laboratory. The Principal User may also be the End User.
End user	The individual who possesses and uses synthetic nucleic acids that they have received from a Customer, Principal User, or another End User.
Synthetic nucleic acid	A single- or double-stranded DNA or RNA fragment produced by nucleic acid synthesis technology.
Sequence of Concern (SOC)	A broad term often used to capture the ideal not just of sequences currently subject to formal regulatory control but any sequence that could be misused to cause significant harm.<br><br>The 2023 updated U.S. government Guidance defines SoCs to include "sequences known to contribute to pathogenicity or toxicity, even when not derived from or encoding regulated biological agents" without further elaboration.<br><br>Importantly, there is currently no agreed-upon list of such sequences but there are many ongoing discussions around the value of creation of such a list. Should such a list one day exist, the IGSC will evaluate its utility in synthesis screening and update this Protocol accordingly.
Benchtop nucleic acid synthesis equipment	Benchtop nucleic acid synthesis equipment sold by Manufacturers that is intended to be used to synthesize nucleic acids for use within a research laboratory or within an institution. While this nucleic acid synthesis equipment may not be small enough to be placed on a benchtop (e.g., it sits on the laboratory floor), it is still considered benchtop equipment if it is sold with the intent that it will be used by researchers individually or in a core facility in an institution.

Appendix B: Customer Screening Tools

Vendor	
AEB Compliance Screening	https://www.aeb.com/en/trade-compliance-management-software.php
Dow Jones Risk & Compliance Solutions	https://www.dowjones.com/professional/risk/
Lexis Nexis Bridger Insight	https://risk.lexisnexis.com/products/bridger-insight-xg
SAP Global Trade Services (GTS)	https://www.sap.com/products/financial-management/global-trade-management.html
Trademo Sanctions Screener	https://www.trademo.com/global-trade-compliance/sanctions-screener

ISO 20688-2:2024 Biotechnology — Nucleic acid synthesis — Part 2: Requirements for the production and quality control of synthesized gene fragments, genes, and genomes

Assessing Risk and Legitimate Use

This standard cannot be reproduced directly due to copyright considerations, but closely mirrors the IGSC's Harmonized Screening Protocol v2.0, including instructions to:

- For flagged orders
    - Conduct follow-up screening
    - Obtain evidence of legitimate use, such as affiliation or publication history
    - Obtain written descriptions of intended use
- Refuse or report orders when follow-up screening does not resolve concerns
- Verify intended use and other information for consistency with other customer activities
- Ensure appropriate biosafety and biosecurity measures in facilities synthesising pathogen or toxin DNA
